# Behavioral and neuroendocrine consequences of social subjugation across adolescence and adulthood

**DOI:** 10.1186/1742-9994-2-7

**Published:** 2005-04-22

**Authors:** Craig F Ferris, Tara Messenger, Ross Sullivan

**Affiliations:** 1 Center for Comparative Neuroimaging, University of Massachusetts Medical School, Worcester, Massachusetts, USA

## Abstract

**Background:**

Social subjugation is a very significant and natural stressor in the animal kingdom. Adult animals defeated and subjugated during establishment of dominance hierarchies or territorial encounters can be highly submissive in future agonistic interactions. While much is know about the biological and behavioral consequences of winning and losing fights in adulthood, little is known about adolescence; a developmental period noted for impulsivity and heightened agonistic behavior. The present studies were undertaken to determine if the behavioral and neuroendocrine consequences of social subjugation are comparable in adolescent versus adult Syrian golden hamsters (*Mesocricetus auratus*). Male siblings were studied from adolescence into adulthood following exposure to counterbalanced episodes of either a benign stressor, i.e., isolation in a novel cage, or the more severe stressor of social subjugation.

**Results:**

As adults, hamsters with a history of social subjugation in adolescence show high levels of aggression toward intruders as compared to siblings subjugated in adulthood. Sibling controls subjugated in adulthood are highly submissive with little or no aggressive behavior. However, when subjugated in adulthood, hamsters with the earlier history of subjugation are no different than their sibling controls, i.e., adult subjugation promotes submissive behavior. Sexual motivation is high in adult hamsters with adolescent subjugation and testosterone levels remained stable over adulthood. In contrast, sibling controls subjugated in adulthood show lower levels of sexual motivation and reduced levels of testosterone. Release of cortisol during agonistic encounters is blunted in animals subjugated in adolescence but not adulthood. Measures of anxiety are reduced in hamsters with adolescent subjugation as compared to their sibling controls.

**Conclusion:**

These data demonstrate a pronounced difference in behavior and neuroendocrinology between adolescent and adult hamsters in their response to social subjugation and suggest adolescence is a resilient period in development.

## Background

Social subjugation is a natural stressor in the animal kingdom with long-term behavioral consequences. Adult male rhesus monkeys that fight for dominance status and lose are relegated to the lowest social rank displaying highly submissive behavior [1]. Social subjugation in adult male talapoin monkeys reduces social activity and sexual behavior even in the absence of dominant conspecifics [2]. Defeated adult mice and rats display less aggressive and more submissive behavior [3-5]. Individually housed adult hamsters will routinely attack and bite an equal or smaller sized intruder placed into their home cage. However, following repeated defeat by a dominant conspecific, a resident hamster will be defensive or fearful of equal sized non aggressive intruders [6-8].

Social subjugation in adult animals can dramatically alter stress and reproductive hormones levels. There are many reports across species that continuous subjugation results in the dysregulation of the stress response resulting in elevated and protracted levels of stress hormone [8-13]. High basal levels of glucocorticoids are associated with a depressed immune system, diminished reproductive function, bone loss, and increased fear, anxiety and depression [14]. So the reduced aggressive and sexual behavior and heightened submission observed in defeated animals may be due to, in part, dysregulation of stress hormone release. The diminution in offensive aggression and sexual activity may result also from a general reduction in circulating levels of testosterone that normally present in subjugated animals as compared to their dominant male conspecifics [1,15-17].

While the behavioral and neuroendocrine consequences of social subjugation in adult animals are well documented, less in know about the effects of aggressive encounters in the adolescent period. Adolescence is defined as a period of pronounced physical, cognitive and emotional growth. This period usually begins just before puberty and ends in early adulthood with sexual maturity, social awareness and independence [18]. The animal reported on here, the Syrian golden hamster (*Mesocricetus auratus*) has a developmental period analogous to adolescence. In the wild, hamsters wean around P-25 (postnatal day 25), leave the home nest, forage on their own, establish nest sites, and defend their territory [19,20]. Hamsters can begin to establish dominance hierarchies as early as P-35 [21], and have a minimal breeding age of 42 days [22]. Androgen levels start to rise between P-28 and P-35 [23,24]. Thus between P-25 and P-42, as hamsters achieve independence from the maternal nest, they increase their weight and size, reach full sexual maturity and reproductive competence and establish social relationships. This period between P-25 and P-42 is designated as adolescence in golden hamsters. Hamsters in the wild are solitary and live in their own isolated burrows [19,20]. Thus, animals studied in the laboratory setting can be individually housed after weaning, an experimental feature that eliminates the confounding variable of group interactions.

Previous work on social subjugation in adolescent hamsters reported unexpected behavioral changes [25]. Male golden hamsters weaned at P-25, were exposed daily to aggressive adults from P-28 to P-42, and tested for aggression as young adults several days later after the cessation of stress. Animals with a history of social subjugation showed a context-dependent alteration in their aggressive behavior. They showed little or no aggression toward intruders of comparable age and size. However, when confronted by a smaller, younger intruder they were exceedingly aggressive, displaying short attack latencies and high number of bites as compared to sibling controls that were not subjugated during adolescence. Given the dire consequences of social subjugation in adulthood, i.e., low social status, submissive behavior, decreased reproductive activity, higher risk for disease, etc. could these hamsters with a history of adolescent subjugation be able to compete for dominant status as adults? The present studies were undertaken to test the hypothesis that adolescence is a resilient development period, immune to the behavioral consequences of repeated social subjugation.

## Results

### Agonistic Behaviors

**Figure 1:**
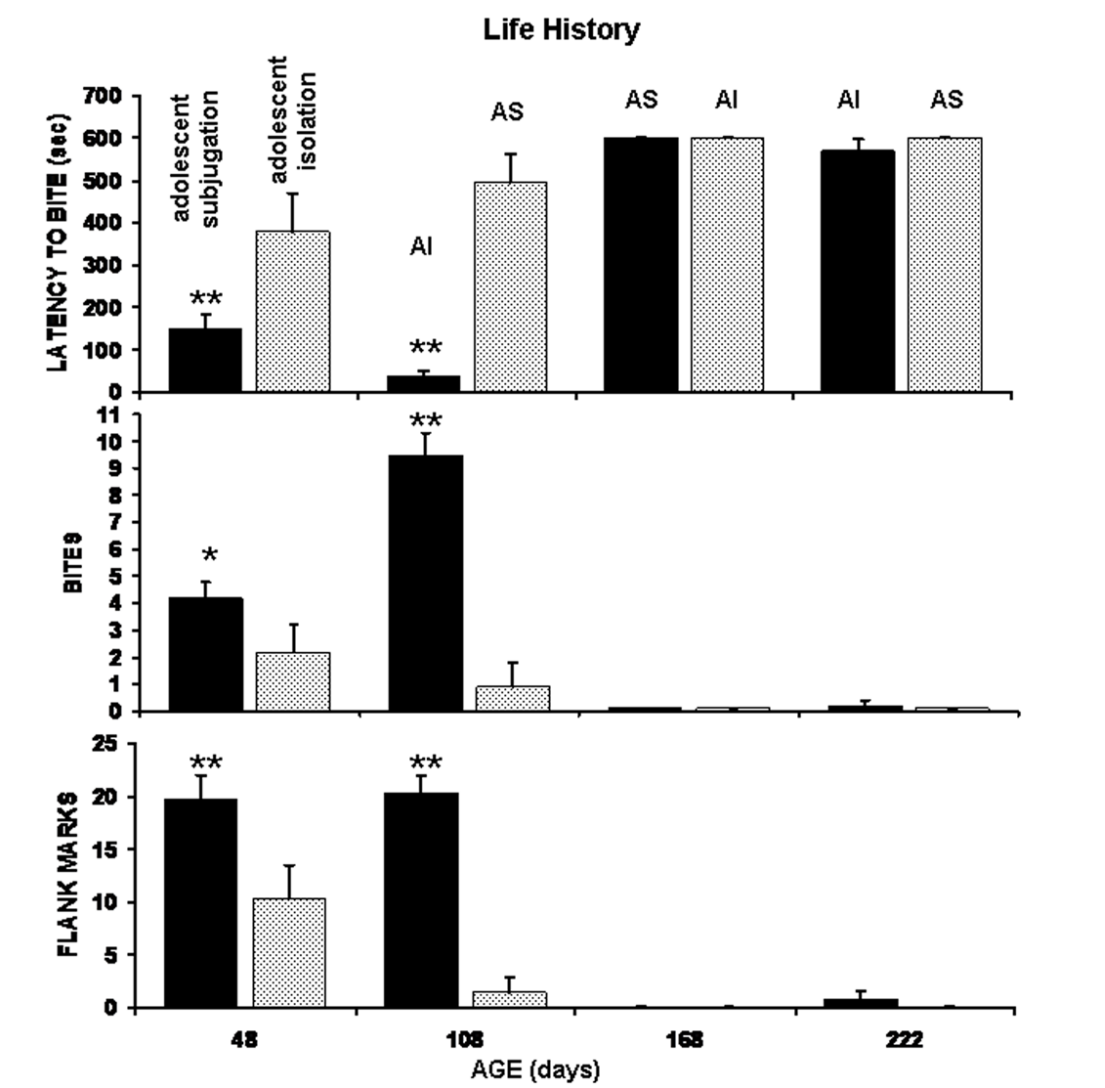
Measures of Aggressive Behavior: Shown are the mean scores (+ SEM) for bite latency, bites and flank marks for hamsters initially subjugated in adolescence (black bar) as compared to their siblings isolated in adolescence (open bar) across a changing social history of adult subjugation (AS) or adult isolation (AI). (* p <.05; ** p < .01).

The effects of social history on biting behavior and flank marking are shown in Figure 1. There was a significant main effect in the latency to bite intruders between AS (adolescent subjugation) and AI (adolescent isolation) hamsters (F_(1,18) _= 23.4, p < 0.01). There was also a significant main effect for repeated test trials across the six month period (F_(3,54) _= 74.15, p < 0.001). When tested as young adults at 48 days of age, AS hamsters on average bite intruders in just over two min while AI hamsters took almost six min to bite (p < 0.01). This difference in bite latency was even more pronounced when animals were tested around 108 days of age. However, at this time AS hamsters were exposed to isolation stress prior to testing, while their AI siblings were subjugated. Average bite latencies were reduced to less than 40 sec in AS hamsters follow adult isolation but delayed by over 8 min in AI hamsters following adult subjugation (p < 0.01). Indeed, the first episode of adult subjugation dramatically inhibited biting behavior regardless of subsequent life histories as noted at 168 and 222 days of age.

The profile of biting attacks complimented the bite latency data. There was a significant main effect in the frequency of biting attacks toward intruders between AS and AI hamsters (F_(1,18) _= 26.9, p < 0.001). There was also a significant main effect for test trials (F_(3,54) _= 37.87, p < 0.001). When tested as young adults at 48 days of age, AS hamsters displayed almost twice as many bites as their AI siblings (p < 0.05). This difference in biting attacks was even more pronounced when hamsters were tested around 108 days of age. Adult subjugation dramatically reduced biting attacks as compared to adult isolation (p < 0.01). This result was confirmed when animals were tested around 168 days of age. Adult subjugation of AS hamsters eliminated biting attacks. Following the first episode of adult subjugation there was never any recovery of biting behavior toward equal sized intruders in either the AS or AI groups.

There was a significant main effect for retreats (F_(1,18) _= 13.7, p < 0.01) and test trials (F_(3,54) _= 27.6, p < 0.001) (data not shown). AS hamsters tested at 48 days of age showed no signs of retreating from intruders as compared to their AI siblings (p < 0.01). At 108 days of age, AI hamsters were highly submissive as compared to their earlier behavior and to the behavior of their AS siblings (p < 0.01). Adult subjugation of AS hamsters also resulted in highly submissive behavior. This submissive behavior following the first episode of adult subjugation persisted through 168 and 222 days of age for both AS and AI groups regardless of life histories.

There was a striking similarity between the behavioral pattern of flank marking and the frequency of biting over the life history of both AS and AI groups. There was a significant main effect for the number of flank marks (F_(1,18) _= 42.6, p < 0.001) and test trials (F_(3,54) _= 44.5 p < 0.001). At 48 days of age, AS hamsters displayed over twice the number of flank marks as their AI siblings (p < 0.01). This high level of flank marking persisted at 108 days of age following the stress of adult isolation; however, their AI siblings for the first time displayed little or no flank marking (p < 0.01). Similarly, AS hamsters, when exposed to their first episode of subjugation as adults ceased to flank mark in the presence of an intruder.

### Locomotor Activity, Anxiety, and Sexual Motivation

**Figure 2:**
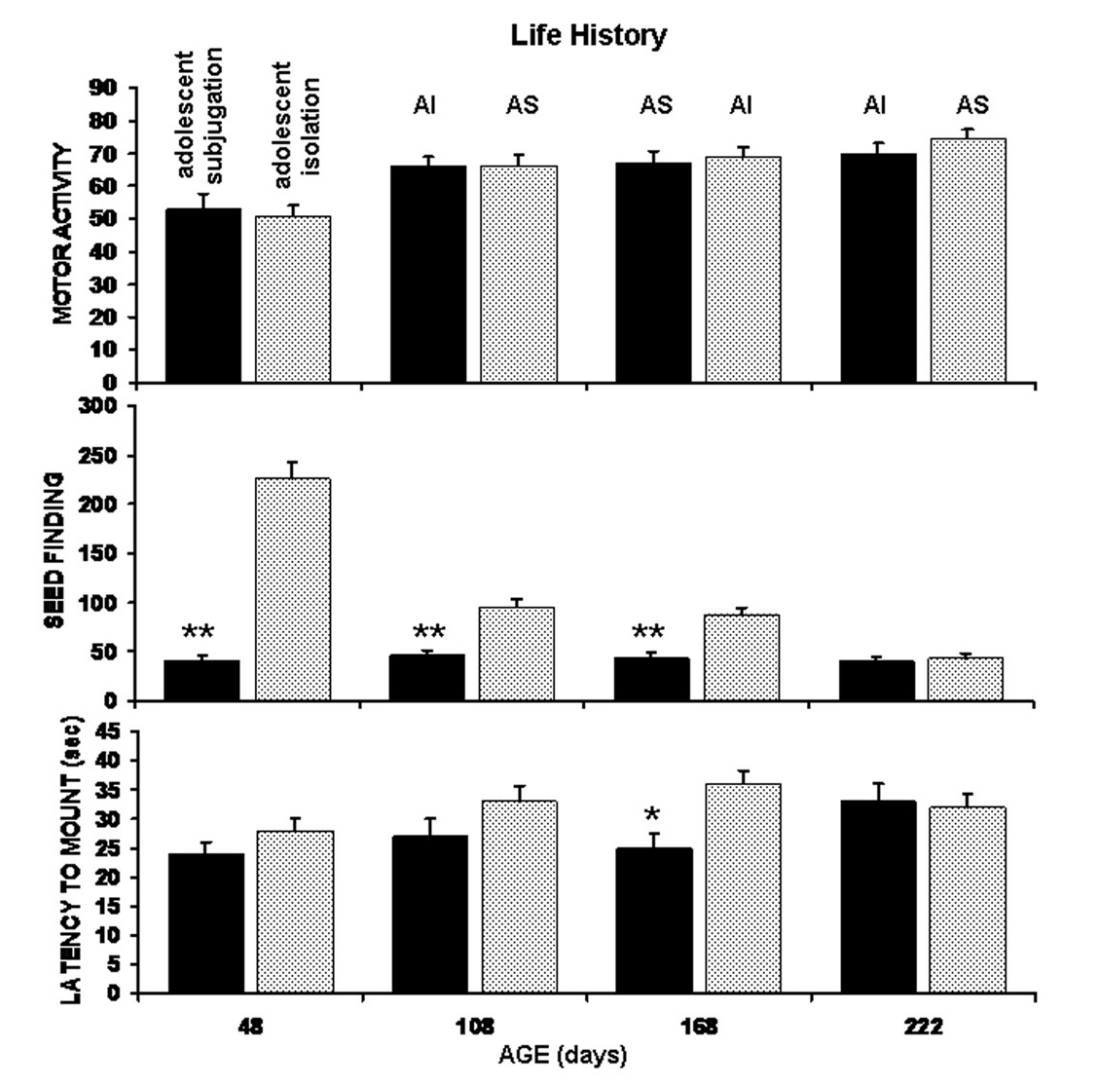
Measures of General Behaviors: Shown are the mean scores (+ SEM) for seed finding (time in seconds), motor activity (number of quadrants traversed in one min) and mount latency for hamsters initially subjugated in adolescence (black bar) as compared to their siblings isolated in adolescence (open bar) across a changing social history of adult subjugation (AS) or adult isolation (AI). (* p <.05; ** p < .01)

Data showing the effect of life history on locomotor activity in an open field, anxiety, and sexual motivation are shown in Figure 2. There was no significant main effect for motor activity (F_(1,18) _= 0.18, p > 0.5); however there was a significant main effect for trials over the six month testing period (F_(3,54) _= p < 0.001). While motor activity was similar for both AS and AI groups, there was a significant increase in activity at the older ages.

Stress-induced anxiety, as measured by the latency to find hidden seeds, was significantly different between the AS and AI groups (F_(1,18) _= 100.6, p < 0.001). There was also a significant main effect on seed finding across test trials (F_(3,54) _= 75.8, p < 0.001). There were no significant differences in seed finding across the life history of AS hamsters. In contrast, AI hamsters took almost four min to find the seeds as compared to about 40 sec for their AS siblings (p < 0.01). At 108 and 168 days of age the latency to find seeds was reduced but still significantly higher than the AS group (p < 0.01). It was not until the final and second adult subjugation that the latency to find seeds was comparable between AS and AI groups.

The latency to mount a receptive female as a measure of sexual motivation was significantly different between AS and AI groups (F_(1,18) _= 7.47, p < 0.05). However, there was no significant difference in mounting latency within each group across test trials (F_(3,54) _= 2.03, p > 0.1). At 168 days of age, AS hamsters, took less time to mount a receptive female than their AI siblings (p < 0.05). At 222 days of age, AS and AI groups showed the same latency in mounting behavior.

### Neuroendocrine Measures

**Figure 3:**
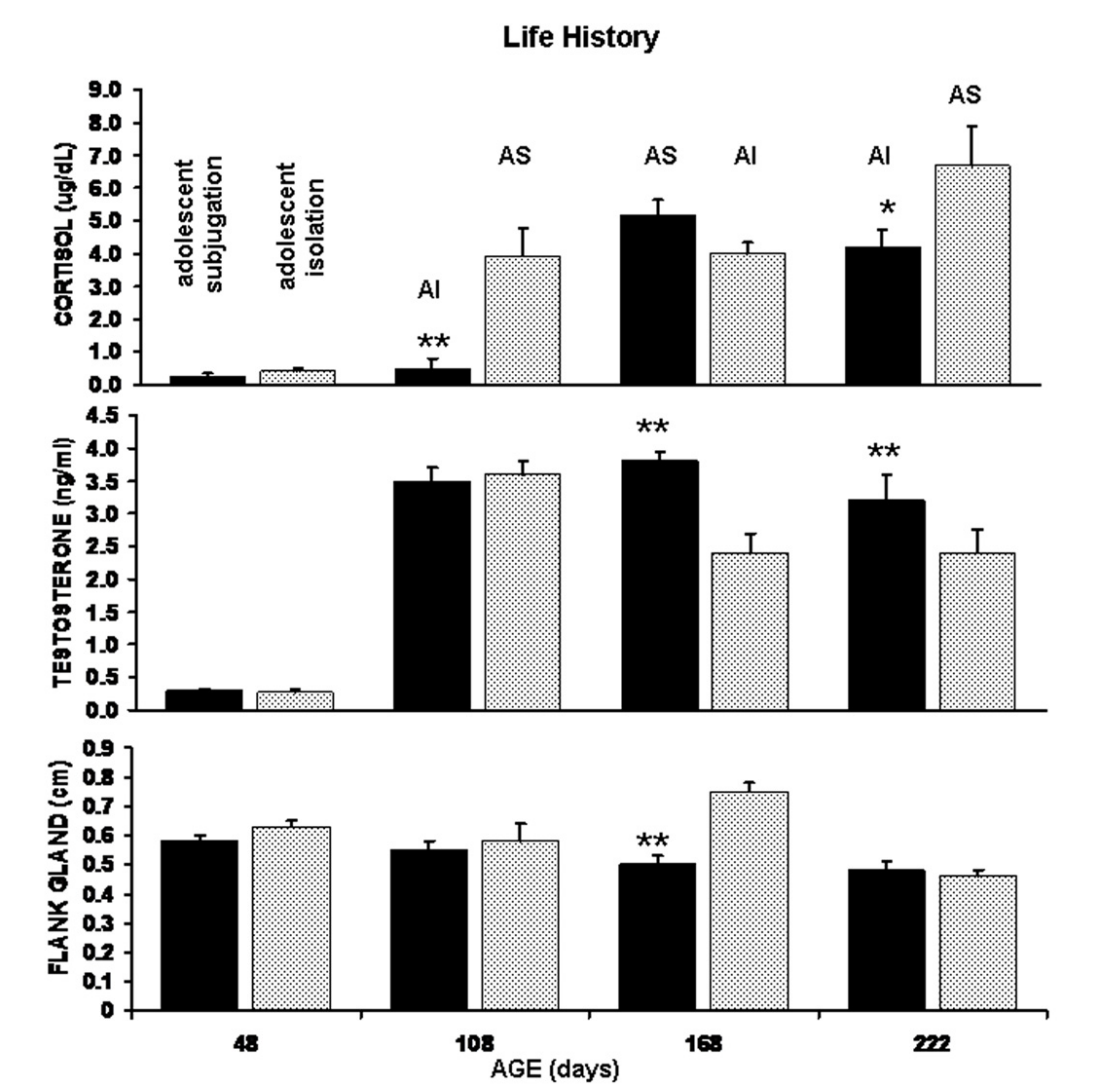
Measures of Steroid Hormone Changes: Shown are the mean plasma levels of cortisol and testosterone and the diameter of the androgen-sensitive flank glands (+ SEM). Measures are collected over a changing social history from hamsters initially subjugated in adolescence (black bar) as compared to their siblings isolated in adolescence (open bar). AS – adult subjugation; AI – adult isolation (* p <.05; ** p < .01)

Data showing changes in blood levels of cortisol, testosterone and the androgen-sensitive flank glands are shown in Figure 3. The levels of cortisol between the AS and AI groups were significantly different (F_(1,18) _= 25.37, p < 0.0001). There was also a change of cortisol levels over time (F_(3,54) _= 9.32, p < 0.01). At 48 days of age both AS and AI siblings showed no appreciable release of stress hormone following an agonistic encounter with an adult intruder. This same blunted or suppressed stress response was observed for AS hamsters when they were tested at 108 days of age against an adult intruder (p < 0.01). In contrast, their AI siblings subjugated as adults showed significantly greater cortisol release following the interaction with an adult intruder. This pronounced difference disappeared at ages 168 after both groups had been exposed to adult subjugation. However, AI hamsters exposed to their second experience of adult subjugation at 222 days of age showed the highest cortisol levels of any sampling period (P < 0.05).

The levels of testosterone between the AS and AI groups were significantly different (F_(1,18) _= 9.43, p < 0.01). There was also a significant change in testosterone across the test trials (F_(3,54) _= 74.1, p < 0.001). At 48 days of age, AS and AI siblings showed comparable levels of testosterone that were significantly lower than those measured at 108 days of age and older. At 108 days of age and older, AS hamsters showed no significant change in testosterone. While AI hamsters showed high levels of testosterone at 108 days of age following adult subjugation, their levels were significantly reduced at 168 and 222 days of age following experiences of isolation and subjugation, respectively (p < 0.01).

The size of the androgen-sensitive flank glands was significantly different between groups (F_(1,18) _= 8.15, p < 0.01) and across test trials (F_(3,54) _= 11.2, p < 0.001). AS siblings presented with the same sized flank glands over the course of their life history. In contrast, their AI siblings showed variable and significantly different sized flank glands across their life history. At 168 days of age their glands were significantly larger than at any age of the AS group's life history (p < 0.01).

## Discussion

The anticipated changes in behavior with social subjugation noted in the literature for adult animals were not apparent in adolescent hamsters. For twelve consecutive days AS hamsters were placed into the home cage of a novel, larger, more aggressive hamster. As predicted, they were threatened and attacked each day over the thirty min encounter. Ten days after the cessation of subjugation the "table was turned" and these AS hamsters, now as young adults, became the residents and novel, larger hamsters became the intruders. In this context, and despite the social history of subjugation, AS hamsters were very aggressive. This display of aggression toward adult intruders was only heightened at three and half months of age as these AS hamsters grew into full maturity. In contrast, their AI siblings were far less aggressive as young adults, avoiding and retreating from adult intruders. When subjugated as fully mature adults, these AI hamsters took on a stable, submissive behavioral profile with no signs of aggression toward adult intruders. Adult subjugation was equally effective in promoting submissive behavior in AS hamsters. While adolescent subjugation favored future aggressive behavior, it did not protect these hamsters from the behavioral consequences of losing fights as adults. When socially subjugated as adults their fate was the same as their AI siblings – a stable, submissive behavioral profile.

Motor activity in the open field was essentially no different between AS and AI siblings over their life history. The latency to mount a receptive female as a measure of sexual motivation was not disrupted in either sibling group; although, there was a modest but significant delay in mounting time by the AI hamsters following their adult subjugation as compared to their AS siblings. The seed finding as a measure of anxiety showed an interesting pattern over the social history of the two sibling groups. AS hamsters are less anxious and attend to finding seeds in their home territory. Their AI siblings are more anxious but after repeated social subjugation in adulthood show the same attention to finding seed. From these observations, it would seem that socially subjugated male hamsters maintain behaviors necessary for survival despite a stable submissive phenotype that would limit their ability to compete for mates.

In an earlier paper, Delville et al. [25] reported social subjugation in adolescent hamsters increased biting attacks toward smaller intruders with reduced generalized aggression toward conspecifics of equal size. The study was designed to determine whether early traumatic stress in the form of social subjugation fostered inappropriate aggressive behavior. Indeed, the short bite latency and excessive number of bites toward non-threatening prepubescent submissive hamsters was judged to be inappropriate as compared to controls with no history of early abuse. However, when control and subjugated animals were tested against adult male intruders the level of biting attacks was very low or absent in both groups. Consequently, offensive aggression was reduced to the less specific behavioral measure of "attacks", or aggressive approaches and not as bite latency and number of bites as reported here. While there are probably many reasons for the high level of biting behavior toward adult intruders in our study versus the relative absence of biting attacks in the other, two possible explanations stand out. First, is the choice of the adult intruder. Delville and colleagues prescreened all male hamsters in adolescence for aggressive behavior. If animals showed any submissive predisposition they were eliminated from the population or used as small prepubescent intruders. Hence, the adult male intruders in their study may have presented as being more aggressive than typical males, discouraging biting attacks from the residents. The second explanation may be the value placed on the defense of the home territory by the resident. In our studies, resident's experienced temporary food deprivation associated with seed finding and access to a receptive female on the days prior to presentation of an unknown adult intruder. Under these circumstances, the level of offensive aggression toward intruders may be heightened as resident's fight to defend a territory perceived to have high reproductive potential and limited resources.

In a more recent study, Wommack and Delville [26] reported hamsters 42 days of age with a history of adolescent subjugation were highly submissive in the home territory of the larger more aggressive hamsters. These results are not surprising, as some level of reduced aggression or submission would be expected when smaller male hamsters are placed into the home cage of larger conspecifics [27]. In contrast, the present study has experimental and control siblings displaying offensive aggression to defend their own territory from adult male intruders. Given the context-dependent nature of aggressive responding it is difficult to compare the data between the two studies.

The anticipated suppression of plasma testosterone and elevation in stress hormone in adult animals following social subjugation did not occur in adolescent hamsters. Early adolescent stress followed by exposure to episodes of social and environmental stressors over adulthood produced an unexpected pattern of steroid hormone release in response to social conflict. The first unanticipated finding was the low plasma levels of testosterone (<0.5 ng/ml) in 48 day old AS and AI siblings following an aggressive encounter with an adult male intruder. Levels for this age are usually 2–4 ng/ml or near maximum for adult animals on long light/dark cycles [24,28]. Indeed, in our own laboratory we measured testosterone levels of 1–2 ng/ml in unstressed P-45 hamsters [29]. Both AS and AI siblings showed aggressive behavior toward the intruders. As noted above, the AS hamsters were far more aggressive than their AI siblings. While golden hamsters do not need testosterone to develop aggressive behavior [21], this steroid hormone augments aggressive responding and favors dominance behavior [30,31].

The low levels of testosterone during transition from adolescence to young adulthood may represent suppression of the hypothalamic-pituitary-gonadal axis caused by the stress of social subjugation or isolation in a novel environment. However, this is unlikely since both AS and AI groups showed normal adult-sized flank glands at 48 days of age. The flank glands are androgen sensitive; their size and pigmentation have been correlated with plasma testosterone levels [31]. Perhaps the low levels of testosterone are transient, decreasing over the 15 min period from start of social interaction to blood sampling. A significant decrease in plasma testosterone following defeat was noted in Siberian dwarf hamsters [32] and guinea pigs [33] within 10–15 min of the social encounter. Huhman and coworkers [8] noted a tendency for hamsters to show lower levels of testosterone 15–20 min after a single defeat but the trend was not significant. This robust suppression of testosterone levels following social conflict in 48 day old hamsters was not unique to their social history since both AS and AI siblings showed the same endocrine response. However, what is common to both social histories is adolescent stress.

Interestingly, fully mature hamsters tested at 108 days of age showed stable plasma levels of testosterone (ca. 3–4 ng/ml) after social conflict despite two opposite agonistic behaviors. AS hamsters were extremely aggressive, while their AI siblings were highly submissive. The endocrine response of the AI hamsters was unexpected since social subjugation in adult rodents reduces androgen levels [8,9]. Indeed, it was only after repeated exposure to episodes of subjugation and isolation stress over adulthood, did AI hamsters show significantly reduced (ca. 2 ng/ml) levels of testosterone that correlated with their submissive behavior. Why this reduction in testosterone was not observed following their first experience of social subjugation is unknown. Perhaps the isolation stress during adolescence provided some "immunity" to future stressors, sparing the hypothalamic-pituitary-gonadal axis. However, this early resilience is lost as hamsters are continuously exposed to chronic episodes of social and environmental stressors over adulthood. In contrast, AS hamsters show stable levels of testosterone over their adult social history. Although they show submissive behavior following subjugation in adulthood their testosterone levels are unchanged following defeat. Unlike their AI sibling, their "immunity" to chronic episodes of social and environmental stressors over adulthood persists.

Instead of the anticipated elevation in cortisol levels associated with social conflict, both AS and AI siblings showed a suppressed cortisol response (<0.3 ug/dL) following an aggressive encounter at 48 days of age. When fully mature at three and half months of age, AS hamsters showed the same blunted stress response following a successful attack on an adult intruder. This high aggression in the face of a blunted cortisol response is not unlike that reported by Halász and coworkers in rats [34]. Following adult subjugation, AI hamsters showed increased cortisol release associated with submissive behavior toward intruders. Their AS siblings showed the same release of cortisol, but only after adult subjugation.

Because of the 7–8 month duration of this study and the logistics of controlling for each developmental time point, there were limitations in the experimental design. Ideally, at each of the four test periods, 48, 108, 168, and 222 a sibling control group without any history of adolescent stress (subjugation or isolation) would have aided in interpreting developmental changes in behavior. However, it was not possible to get the required number (n = 40) male siblings from the same dams that provided AS and AI groups. This deficiency not withstanding, the behavioral results following adolescent subjugation and that following adult subjugation corroborate much of the previous work on hamsters using experimental animals with no history of winning and losing fights [6-8]. What are unique about these studies are the developmental consequences of repeated agonistic encounters in which the out come of winning or losing is predetermined.

## Conclusion

Adolescence is a time of enhanced physical growth, driven, in part, by rising testosterone levels culminating in sexual maturity. Around P-25 male hamsters leave the maternal nest to forage for food and live alone in their own burrow. Losing fights might be very common for adolescent hamsters because they have not developed fully the size, strength and social behaviors to compete for food, mates and territory. If the results of repeated defeat in adolescence were a stable submissive phenotype toward all competitors then these hamsters might never have the opportunity to mate. The data presented here show that adult hamsters with a history of subjugation in adolescence can be very aggressive toward male conspecifics. Hence, adolescence may be a resilient period in development, protecting animals until they reach adulthood.

## Methods

The breeding stock of Syrian golden hamsters was obtained from Harlan Sprague-Dawley Laboratories (Indianapolis, IN). All animals were housed individually in Plexiglas cages (24 cm × 24 cm × 20 cm), maintained on a reverse light:dark cycle (14:10; lights on at 19:00 hr) and provided food and water *ad libitum*. All animals were acquired and cared for in accordance with the guidelines published in the *Guide for the Care and Use of Laboratory Animals *(National Institutes of Health Publications No. 85-23, Revised 1985). The protocols used in this study were in compliance with the regulations of the Institutional Animal Care and Use Committee at the University of Massachusetts Medical School.

**Figure 4:**
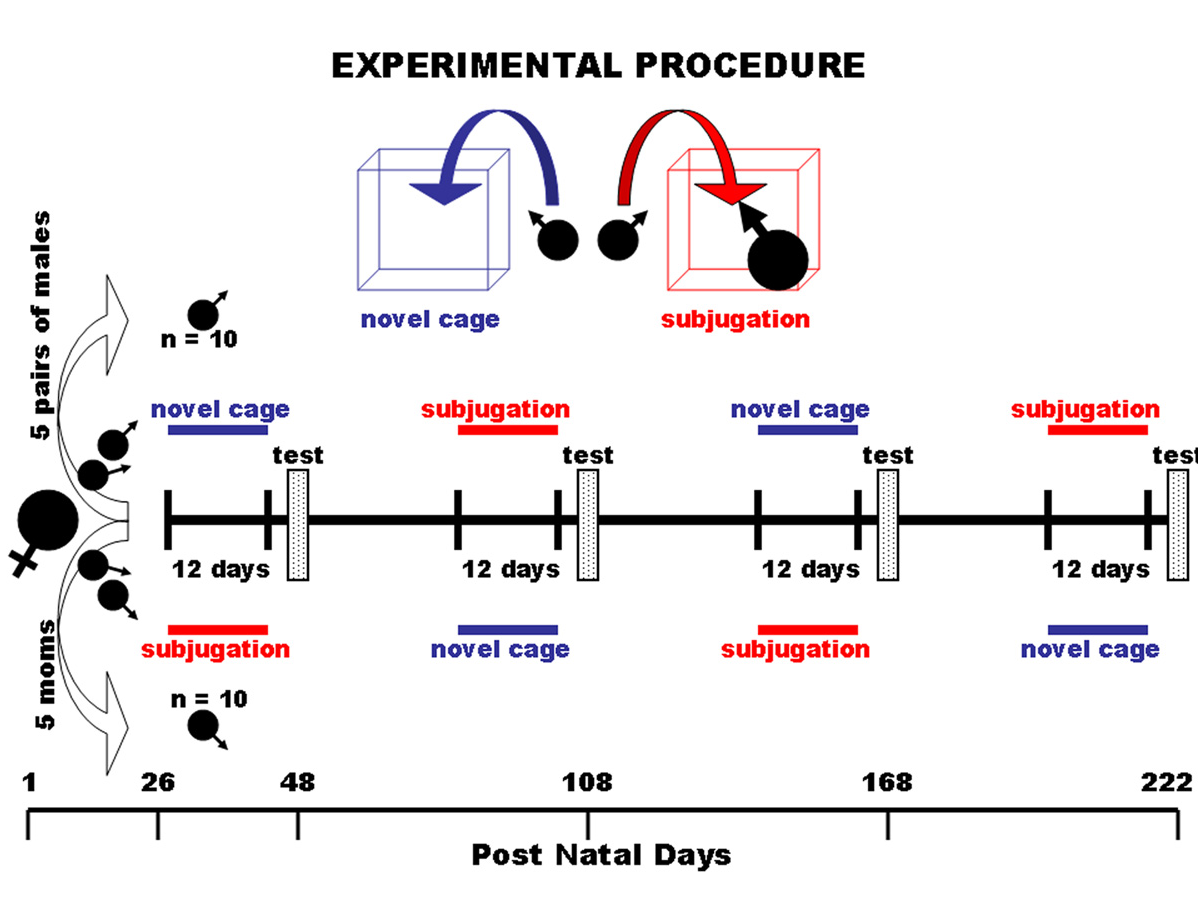
Schematic Diagram of Experimental Procedure: Shown are daily schedules of subjugation stress (red, subjugation) and isolation stress (blue, novel cage) from birth to postnatal day 222 for two groups of 10 hamsters each, sampled from 5 dams. Ten days after the cessation of each stress paradigm there was a three day test period (test) during which hamsters were screened for behavior and bleed for steroid hormone measures.

Female golden hamsters were bred in the animal facility at the University of Massachusetts Medical School. On P-23 male hamsters from each litter were individually housed. A diagram of the experimental procedure is provided in Fig 4. Four male subjects were sampled from each of five dams. These four were divided equally between either a adolescent subjugation (AS) group or adolescent isolation (AI) group. On P-26, the AS group was exposed to the stress of threat and attack by placing them into the home cage of a novel larger, experienced fighter for 30 min/day for 12 consecutive days while the AI group was exposed to the stress of a novel environment by placing them into a new clean cage for 30 min/day for 12 consecutive days. Handling and exposure of hamsters to a novel environment is a stressor as measured by release of cortisol [35]. Hence, these AI siblings control for the daily exposure to a novel environment of their AS siblings. It should be noted, hamsters exposed to subjugation have few if any physical injuries. Biting attacks very seldom cause in wounds.

On P-48, ten days after the cessation of stress, animals were tested and scored for a battery of behaviors over three consecutive days. As young adults these animals were again exposed to the stress of threat and attack of a large experienced fighter or isolation in a novel environment; however, the stressors were reversed. AS animals exposed to social subjugation during adolescence were now exposed to isolation while AI animals exposed to isolation in adolescence were exposed to social subjugation. These studies were begun ca. three months of age because age-dependent increases in aggression are stabile at this time [21]. On P-108, ten days after the cessation of stress, animals were again tested and scored for a battery of behaviors. The procedure was repeated twice more at approximately two month intervals for each group of animals reversing the stressor at each time.

All behavioral tests were performed during the first four hrs of the dark phase under dim red illumination, video taped and scored by an independent observer blind to the history of the animals. Screening began with seed finding a model for screening anti-anxiety drugs in golden hamsters [36]. Briefly, hamsters are deprived of food overnight. The following day they are exposed to the additional stress of being taken from their home cage and placed in a novel environment for a few minutes. During their absence from the home cage, sunflower seeds are hidden under the bedding in one of the corners. When returned to the home cage, hamsters routinely scramble along the walls for 3–4 min before settling down, locating and eating the seeds. However, animals treated with the traditional anxiolytics e.g., chlordiazepoxide, fluoxetine or buspirone find seeds in less than 20 sec. [36]. Seed finding was scored as the latency to find and eat or pouch a single seed.

Following seed finding, hamsters were tested for a sexual motivation by introducing a receptive, estrus female into their home cage. Females would routinely assume the lordotic posture and males were timed for their latency to mount and first intromission. Intromission was characterized by a constant rate of pelvic thrusting. On the second day animals were tested for general motor activity in an "open field." Animals were placed into a large clean Plexiglas cage (48 × 32 × 40 cm) devoid of any bedding. This open field was delineated into equal quadrants by tape on the underside of the cage. Animals were scored for motor activity by counting the number of quadrants traversed in 1 min.

On the third behavioral screening day, animals were tested for agonistic behavior in a resident/intruder paradigm. A male intruder of approximately the same size, weight and age was introduced into the home cage of the test animal and the resident scored for latency to bite the intruder, total number of bites, contact time, retreats and flank marks over a 10 min test period as previously described [37]. Flank marking is a form of olfactory communication in which a hamster arches its back and rubs pheromone producing flank glands against objects in the environment [38]. Flank marking frequency is greatly enhanced during aggressive encounters and is particularly robust in dominant animals initiating and winning fights [39].

Immediately after the end of resident/intruder encounter animals were lightly anesthetized with isoflurane and a venous blood sample of ca. 0.3 – 0.4 ml was collected by orbital eye bleed from the resident. The introduction of the hamster to the isoflurane followed by the sampling took less than 1 min. The samples was heparinized, centrifuged and assayed for testosterone and cortisol using a solid phase ^125^I radioimmunoassay (ICN Biomedical, Inc. Costa Mesa, CA). A total of four blood samples were collected from each animal over the 8–9 month study.

The data for all measures from both AS and AI groups over the four test periods were analyzed with a repeated measures two-way ANOVA followed by Newman-Keuls for post hoc comparisons.
